# Awareness of Anti-adhesive Barriers Among Surgeons in Saudi Arabia

**DOI:** 10.7759/cureus.57942

**Published:** 2024-04-09

**Authors:** Rahaf M Alharbi, Ahmed M Almutairi, Kholod K Alsharari, Wejdan K Almarwani, Abdulrahman B Hussamuldin, Fahad M Alsaadi, Wedyan M Alhazmi

**Affiliations:** 1 College of Medicine, Almaarefa University, Riyadh, SAU; 2 College of Medicine, University of Jeddah, Jeddah, SAU; 3 College of Medicine, Jordan University of Science and Technology, Irbid, JOR; 4 Department of General Surgery, King Fahad General Hospital, Jeddah, SAU

**Keywords:** adhesion prevention, post-operative adhesions, saudi arabia, knowledge, surgeons, laparotomy, anti-adhesive barriers, anti-adhesive agents, adhesion

## Abstract

Introduction

Post-operative adhesions present a number of difficulties, including intestinal obstruction and infertility, and they frequently require readmission due to adhesion-related problems. Notwithstanding these ramifications, there are surprisingly few thorough national surveys that address surgeons' awareness of adhesives. By assessing Saudi surgeons' knowledge of post-operative adhesions and their use of anti-adhesive medications, this study aims to close this knowledge gap.

Methods

This study is a cross-sectional observational research study aimed at Saudi Arabian surgeons utilizing a self-administered, semi-structured online questionnaire. The questionnaire was distributed to participants via social media and in-person email using basic random selection. It included adhesion morbidity and prevalence, pre-operative informed consent issues, adhesion preventive viewpoints, and anti-adhesive chemical use.

Results

There were 111 participants in total, of 41% were experienced surgeons with more than five years of experience. According to the survey, the majority of surgeons occasionally employed anti-adhesive compounds, especially during laparotomies (28%), and 38% never used them during laparoscopies. The study found that participants varied in what they informed patients regarding adhesion complications: 25% of participants informed 5%-10% of the patients about the possible adhesion complications in laparotomy procedures, whereas 26% of participants informed 10-25% of the patients in laparoscopic procedures. Compared with their specialist peers, general surgeons agreed more on the clinical significance of adhesions and prevention. Notably, three-quarters of participants were unclear about when to use anti-adhesive compounds.

Conclusion

While acknowledging the clinical significance of post-operative adhesions and recognizing the potential for prevention, most surveyed surgeons did not include adhesions as a post-operative complication in informed consent. The study underscores a belief in the efficacy of anti-adhesives yet reveals a widespread lack of clarity regarding specific indications for their use. Recommendations include implementing educational sessions during surgical training to heighten awareness of adhesions as a major post-operative complication and to encourage the appropriate utilization of available barriers and pharmacological anti-adhesive products.

## Introduction

Post-operative adhesions, which are fibrous connections formed after abdominal and pelvic surgeries, are a major cause of intestinal obstruction and infertility. Post-operative adhesions were classified into two classes: de novo adhesions, which form at a site that is not initially an operation site, and adhesions re-formation, which are adhesions that develop at sites of adhesiolysis [1]. The frequency of occurrence of adhesions after abdominal surgeries was studied by Parker et al. in a follow-up study that included more than 12 thousand patients. After a 10-year interval, 32.6% of patients who underwent lower abdominal surgeries were readmitted for potential adhesions-related complications. The adhesion frequency varies according to the primary operation site, with the rectum and colon being the most common operation sites [2]. A review article reported that adhesions are the underlying cause of about 32% of intestinal obstruction. Yet, most adhesive intestinal obstructions can be treated conservatively and only about 15% of cases might need surgical intervention [3]. 

The mechanisms of post-operative adhesion pathophysiology are thought to be due to hypoxic insults and oxidative stress that can lead to disruption of mesothelial cell functions, fibrosis of the peritoneum, and adhesion formation. Post-operative inflammatory processes also play a role in the pathogenesis of adhesions. A study suggested a decrease in fibrinolytic activity in peritoneal covering with adhesions compared to those without adhesions [4]. The predisposing factors contributing to adhesion formation include types of surgery, for instance, the incidence of post-operative adhesions is generally higher in upper abdominal surgery (93%) compared to lower abdominal surgery (67%) [3]. Laparoscopic surgeries involve fewer manipulations and small incisions than laparotomy and thus were hypothesized to be less adhesion evoking [5]. Nevertheless, using CO_2_ can induce hypoxia and acidosis, which lead to ischemia and subsequent fibrosis and adhesions [6-8]. Materials used during surgeries, like gloves, gauze, and suture materials, were another contributing factor. Foreign body granuloma was reported in 26% of patients who underwent different abdominal surgeries [9]. There is genetic susceptibility to adhesions, including polymorphism in the gene encoding for plasminogen activator inhibitor-1. Other factors that can increase the susceptibility to adhesions include some drugs like anti-parkinsonian, oral hormonal therapy, and alcohol [6,10].

Although careful surgical technique (ensuring sufficient hemostasis, minimizing surgical material remnants, and minimizing manipulation) can help prevent adhesion formation, anti-adhesive products must be used to minimize adhesion risk to the lowest [11,12]. Anti-adhesives include pharmacological and barrier techniques; materials used in these anti-adhesive products include hyaluronan, cellulose, chondroitin, and polylactic acid. The Food and Drug Administration (FDA) approved three barrier anti-adhesive adjuncts to be used in the United States: the first is Interceed, an oxidized cellulose that was approved for use in open surgeries like laparotomy and some gynecological operations. The second FDA-approved product is Seprafilm, a combination of hyaluronic acid and carboxymethylcellulose. The use of Seprafilm in gynecological operations decreased the incidence of adhesions to the uterus by 36%. The third adjunct is the icodextrin solution, which prevents adhesions after laparoscopic gynecological surgeries. In the assessment studies, this barrier adjunct decreased the incidence of adhesions by 9% [13-15]. Many barrier anti-adhesives may fail to adhere to wet surfaces of the internal organs. Hence, a study by Cui et al. invested in a new modality of using a negatively charged adjunct (Janus Hydrogel), which provides better adherence and good anti-adhesive properties in animal models [16]. Due to the observed lack of national surveys that take an in-depth look at the anti-adhesive knowledge and the utmost importance of this issue, we conducted this study aiming to assess knowledge of post-operative adhesions and anti-adhesive use among Saudi surgeons.

## Materials and methods

Study design, participants, and setting

The study was an observational cross-sectional design conducted between May and October 2023 among surgeons in the Kingdom of Saudi Arabia, excluding non-surgeons. 

The sample size was calculated using the formula N = Z^2 * p * (1 - p) / d^2, where N represents the sample size, Z is the Z statistic corresponding to the desired confidence level, p is the expected prevalence or proportion, and d is the desired precision. Given a Z value of 1.96 for a 95% confidence level, a precision of 5% (0.05), and an expected proportion (p) of 50% (0.50), the minimum required sample size was determined to be 385.

Data collection tool

A preliminary pilot study involving a selected group of surgeons was conducted to assess the questionnaire's clarity and understandability. Participant feedback from this preliminary phase was incorporated into the final survey, involving reorganizing the sequence of questions to improve the flow and logical progression of the questionnaire and changing the formatting to enhance readability and comprehension for participants. 

An online, semi-structured, self-administered English questionnaire was distributed in a Google Form to surgeons in person and via social media. The data was collected through simple random sampling. The questionnaire was adopted from a previous study [17]. It contains six sections; the first one evaluates if the respondent is a surgeon or not, the type of surgeon (general, specialized, trainee, other), and years of experience. The second section includes the prevalence and morbidity of adhesions. The third section is informed consent. The fourth section is the opinion on adhesion prevention. The fifth section is the opinion on anti-adhesive agents. The sixth section is the use of anti-adhesive agents. 

Analysis plan

Data analysis will be performed using IBM SPSS Statistics for Windows, Version 27 (Released 2020; IBM Corp., Armonk, New York, United States). Categorical data was presented in frequencies and percentages. A knowledge score was calculated after coding the scale from strongly disagree to strongly agree, with 0 to 4. The Knowledge total score was used to compare surgeon specialties using the Kruskal-Wallis test. The Fisher exact test was used to compare knowledge between different surgeon specialties. A p-value of less than 0.05 was set as the threshold for statistical significance.

Ethical approval 

The study approval was obtained from the Institutional Review Board of Almaarefa University (IRB No: IRB23-050). The online questionnaire contained a consent form detailing the rights of the participants, including voluntary participation, anonymity, confidentiality, and a right to withdraw without justification. 

## Results

One hundred eleven participants were in the study, with the majority being specialized surgeons (41%), and most had more than five years of experience (38%) (Table 1).

**Table 1:** Demographic characteristics of the participants *Specialized surgeons include oncologic surgeons, vascular surgeons, gastrointestinal surgeons, pediatric surgeons, trauma surgeons, and OB/GYN *Other includes any other specialized surgeons whose specialties are not listed above.

Characteristic	N = 111
Specialty	
General Surgeon	31 (28%)
Specialized surgeon*	45 (41%)
Trainee	30 (27%)
Other*	5 (4.5%)
Work experience	
One year or less	29 (26%)
2-3 Years	23 (21%)
4-5 Years	17 (15%)
More than five years	42 (38%)

The majority had a neutral opinion regarding clinical importance (33%) and believed in adhesion prevention (34%). General surgeons expressed the highest disagreement (48%), while specialized surgeons showed the lowest disagreement (13%) regarding the clinical importance of adhesion. Regarding belief in adhesion prevention, general surgeons and trainees exhibited higher levels of disagreement (23% and 13%, respectively), while specialized surgeons demonstrated lower disagreement (6.7%) (Table 2). 

**Table 2:** Beliefs about clinical importance and the prevention activity of adhesion * surgeons include oncologic surgeons, vascular surgeons, gastrointestinal surgeons, pediatric surgeons, trauma surgeons, and OB/GYN *Other includes any other specialized surgeons whose specialties are not listed above.

Characteristic	Overall, N = 111^1^	General surgeon, N = 31^1^	Other, N = 5^1^	Specialized surgeon, N = 45^1^	Trainee, N = 30^1^
Adhesions are not of clinical interest					
Strongly disagree	30 (27%)	15 (48%)	1 (20%)	6 (13%)	8 (27%)
Disagree	24 (22%)	8 (26%)	1 (20%)	9 (20%)	6 (20%)
Neutral	37 (33%)	5 (16%)	1 (20%)	17 (38%)	14 (47%)
Agree	9 (8.1%)	1 (3.2%)	2 (40%)	4 (8.9%)	2 (6.7%)
Strongly agree	11 (9.9%)	2 (6.5%)	0 (0%)	9 (20%)	0 (0%)
You do not believe in adhesion prevention					
Strongly disagree	14 (13%)	7 (23%)	0 (0%)	3 (6.7%)	4 (13%)
Disagree	28 (25%)	11 (35%)	2 (40%)	11 (24%)	4 (13%)
Neutral	38 (34%)	7 (23%)	1 (20%)	14 (31%)	16 (53%)
Agree	21 (19%)	5 (16%)	2 (40%)	11 (24%)	3 (10%)
Strongly agree	10 (9.0%)	1 (3.2%)	0 (0%)	6 (13%)	3 (10%)
^1^n (%)					

Approximately 25% and 38% of surgeons never used anti-adhesive agents in laparotomies and laparoscopies, while 28% and 23% used for single time in laparotomies and laparoscopies, respectively. The majority of general surgeons never use it in laparotomies (29%) not laparoscopies (65%) while the majority of specialized surgeons used once in laparotomies (38%) and laparoscopies (33%), respectively (Table 3).

**Table 3:** Use of anti-adhesive agents in laparotomy and laparoscopy among surgeon *surgeons include; Oncologic surgeon, Vascular surgeon, Gastrointestinal surgeon, Pediatric surgeon, Trauma surgeon, OB/GYN *Other include: any other specialized surgeons whose specialties are not listed above.

Characteristic	N	Overall, N = 111^1^	General surgeon, N = 31^1^	Other, N = 5^1^	Specialized surgeon, N = 45^1^	Trainee, N = 30^1^	p-value^2^
How many times are anti-adhesive agents in laparotomies used by you or your colleagues in your hospital	111						0.020
Never used		28 (25%)	9 (29%)	3 (60%)	4 (8.9%)	12 (40%)	
One time		31 (28%)	5 (16%)	0 (0%)	17 (38%)	9 (30%)	
2 times		20 (18%)	7 (23%)	1 (20%)	7 (16%)	5 (17%)	
3 times		15 (14%)	3 (9.7%)	0 (0%)	9 (20%)	3 (10%)	
4 times		2 (1.8%)	0 (0%)	0 (0%)	2 (4.4%)	0 (0%)	
More than 5 times		15 (14%)	7 (23%)	1 (20%)	6 (13%)	1 (3.3%)	
How many times are anti-adhesive agents in laparoscopic used by you or your colleagues in your hospital	111						<0.001
Never used		42 (38%)	20 (65%)	3 (60%)	8 (18%)	11 (37%)	
One time		26 (23%)	2 (6.5%)	1 (20%)	15 (33%)	8 (27%)	
2 times		21 (19%)	0 (0%)	0 (0%)	12 (27%)	9 (30%)	
3 times		11 (9.9%)	5 (16%)	0 (0%)	6 (13%)	0 (0%)	
4 times		3 (2.7%)	1 (3.2%)	0 (0%)	1 (2.2%)	1 (3.3%)	
More than 5 times		8 (7.2%)	3 (9.7%)	1 (20%)	3 (6.7%)	1 (3.3%)	
Do you believe the use of anti-adhesive barriers will increase in the future	111						0.5
No		17 (15%)	7 (23%)	0 (0%)	5 (11%)	5 (17%)	
Yes		94 (85%)	24 (77%)	5 (100%)	40 (89%)	25 (83%)	
^1^n (%) ^2^Fisher's Exact Test							

Regarding patients informing about adhesions or adhesion-related morbidity as potential complications after both laparotomy and laparoscopic surgeries, 25% of surgeons informed 5-10% of the patient about adhesion problems after laparotomic procedure and (26%) of surgeons informed 10%-25% of the patients in laparoscopic procedures. Notably, a considerable portion of general surgeons (9.7%) and trainees (13%) reported informing less than 5% of patients, while specialized surgeons demonstrated a notably lower proportion (8.9%). In contrast, general surgeons (39%) were more inclined to inform virtually all patients about adhesions, whereas other specialties exhibited lower percentages. For laparoscopic surgeries, the communication patterns also varied significantly (p = 0.031). General surgeons (16%) and trainees (20%) tended to inform less than 5% of patients, whereas specialized surgeons (11%) demonstrated a lower frequency. These findings underscore the diversity in healthcare professionals' communication practices regarding adhesions and related complications, emphasizing the need for standardized communication strategies (Table 4).

**Table 4:** Frequency of patients informed about adhesion complications after laparotomy and laparoscopy

Characteristic	N	Overall, N = 111^1^	General surgeon, N = 31^1^	Other, N = 5^1^	Specialized surgeon, N = 45^1^	Trainee, N = 30^1^	p-value^2^
How many patients do you inform about adhesions or adhesion-related morbidity as a possible complication after laparotomy surgery	111						<0.001
<5%		11 (9.9%)	3 (9.7%)	0 (0%)	4 (8.9%)	4 (13%)	
10%-25%		20 (18%)	1 (3.2%)	1 (20%)	11 (24%)	7 (23%)	
25%-50%		17 (15%)	3 (9.7%)	0 (0%)	9 (20%)	5 (17%)	
5-10%		28 (25%)	8 (26%)	0 (0%)	13 (29%)	7 (23%)	
50%-75%		8 (7.2%)	4 (13%)	0 (0%)	1 (2.2%)	3 (10%)	
None		9 (8.1%)	0 (0%)	2 (40%)	4 (8.9%)	3 (10%)	
Virtually all		18 (16%)	12 (39%)	2 (40%)	3 (6.7%)	1 (3.3%)	
How many patients do you inform about adhesions or adhesion-related morbidity as a possible complication after laparoscopic surgery	111						0.031
<5%		16 (14%)	5 (16%)	0 (0%)	5 (11%)	6 (20%)	
10%-25%		29 (26%)	2 (6.5%)	0 (0%)	15 (33%)	12 (40%)	
25%-50%		13 (12%)	4 (13%)	0 (0%)	7 (16%)	2 (6.7%)	
5-10%		24 (22%)	7 (23%)	1 (20%)	10 (22%)	6 (20%)	
50%-75%		3 (2.7%)	1 (3.2%)	0 (0%)	1 (2.2%)	1 (3.3%)	
None		13 (12%)	5 (16%)	2 (40%)	5 (11%)	1 (3.3%)	
Virtually all		13 (12%)	7 (23%)	2 (40%)	2 (4.4%)	2 (6.7%)	
^1^n (%) ^2^Fisher's Exact Test							

About 32.5% disagreed with the non-clinical importance of adhesion, 42.2% strongly agreed with the beliefs of the beneficial effects of adhesions, and 37.3% agreed with the non-effectiveness of adhesiolysis for pain complaints (p < 0.05). About 35% of our participants agreed that adhesion prevention should be applied with specific indications; however, three-quarters experienced a lack of clarity about when to use an anti-adhesive agent. Participants' preferences regarding the application of adhesion prevention in abdominal operations revealed distinctions based on different indications. For instance, those strongly disagreeing scored an average of 34% for applying prevention in all operations and 33.6% for applying prevention only in certain indications. Approximately 34.4% strongly agreed that laparoscopic surgery causes fewer adhesions than open surgery, and 38.5% strongly agreed that meticulous surgical technique reduces adhesions. Those who strongly disagreed that the position of the mesh and the use of electrocautery cause fewer adhesions scored lower than those strongly agreeing individuals. About 41.3% strongly agreed that costs do not outweigh the possible benefits of antiadhesive agents (p<0.05). Approximately 42.3% preferred using a locally acting anti-adhesive agent over a systematic agent (p<0.05). Regarding informing patients about adhesions-related morbidity after laparotomy and laparoscopic surgery, 36% and 37.5% did not inform any patient (p>0.05) (Table 5).

**Table 5:** Predictors of adhesion knowledge

Characteristic	N = 111^1^	p-value^2^
Adhesions are not of clinical interest		<0.001
Strongly disagree	33.6±5.1	
Disagree	32.5±5.6	
Neutral	35.3±5.1	
Agree	36.9±6.5	
Strongly agree	42.5±6.7	
Adhesions have more beneficial than detrimental effects		<0.001
Strongly disagree	31.1±4.8	
Disagree	34.1±5.4	
Neutral	34.4±4.6	
Agree	36.5±6.0	
Strongly agree	42.2±6.6	
Adhesiolysis for complaints of pain is not effective		<0.001
Strongly disagree	32.4±8.1	
Disagree	32.7±5.3	
Neutral	35.1±4.5	
Agree	37.3±5.2	
Strongly agree	42.5±5.3	
You do not believe in adhesion prevention		0.068
Strongly disagree	32.9±7.1	
Disagree	33.3±5.5	
Neutral	36.0±5.9	
Agree	36.1±5.0	
Strongly agree	37.6±7.7	
You would like to apply adhesion prevention in all abdominal operations		0.5
Strongly disagree	34.0±11.5	
Disagree	32.5±6.2	
Neutral	35.5±6.1	
Agree	35.9±4.9	
Strongly agree	36.1±4.8	
You would like to apply adhesion prevention only in certain indications		0.041
Strongly disagree	33.6±8.3	
Disagree	31.3±6.2	
Neutral	35.3±6.5	
Agree	36.5±4.2	
Strongly agree	35.4±7.0	
Laparoscopic surgery causes fewer adhesions than open surgery		0.006
Strongly disagree	34.4±6.2	
Disagree	26.9±7.4	
Neutral	34.6±6.3	
Agree	35.9±5.5	
Strongly agree	36.9±4.4	
Meticulous surgical technique tissue handling avoiding gauzes reduces adhesions		0.002
Strongly disagree	28.5±6.4	
Disagree	30.4±6.4	
Neutral	34.4±4.6	
Agree	35.3±5.9	
Strongly agree	38.5±6.4	
The extraperitoneal mesh position causes fewer adhesions than the intraperitoneal mesh position		<0.001
Strongly disagree	28.3±5.7	
Disagree	28.4±6.6	
Neutral	34.6±4.2	
Agree	36.2±5.6	
Strongly agree	40.0±6.0	
A coated mesh causes fewer adhesions than an uncoated mesh		0.050
Disagree	31.6±6.7	
Neutral	33.8±5.1	
Agree	35.9±5.9	
Strongly agree	38.1±7.2	
Electrocautery causes fewer adhesions		<0.001
Strongly disagree	27.4±6.1	
Disagree	32.2±5.7	
Neutral	34.8±4.1	
Agree	38.9±5.6	
Strongly agree	40.8±7.9	
Less intraperitoneal suture material reduces adhesions		<0.001
Strongly disagree	23.0±1.7	
Disagree	29.8±6.2	
Neutral	34.2±4.7	
Agree	36.1±4.9	
Strongly agree	41.7±6.4	
You don’t believe in anti-adhesive agents		0.15
Strongly disagree	32.0±5.4	
Disagree	33.1±5.4	
Neutral	35.6±5.5	
Agree	38.5±6.3	
Strongly agree	35.1±9.8	
You experience a lack of clarity about when to use an anti-adhesive agent		0.006
Strongly disagree	27.3±6.2	
Disagree	32.0±6.8	
Neutral	34.5±4.3	
Agree	36.3±6.2	
Strongly agree	38.9±6.8	
You prefer using a locally acting anti-adhesive agent over an agent that acts throughout the whole abdomen		0.002
Strongly disagree	28.4±5.0	
Disagree	32.3±6.0	
Neutral	34.5±4.4	
Agree	36.5±6.2	
Strongly agree	42.3±9.2	
You think the costs do not outweigh the possible benefits of antiadhesive agents		0.005
Strongly disagree	33.0±9.0	
Disagree	31.0±6.1	
Neutral	34.5±4.4	
Agree	37.0±6.2	
Strongly agree	41.3±7.2	
How many patients do you inform about adhesions or adhesion-related morbidity as a possible complication after laparotomy surgery		>0.9
None	36.0±6.5	
<5%	35.0±4.7	
5-10%	34.8±4.5	
10%-25%	34.5±7.3	
25%-50%	36.1±7.9	
50%-75%	35.1±6.2	
Virtually all	34.8±6.0	
How many patients do you inform about adhesions or adhesion-related morbidity as a possible complication after laparoscopic surgery		0.2
None	37.5±5.9	
<5%	36.5±3.2	
5-10%	35.1±5.0	
10%-25%	33.7±7.5	
25%-50%	37.0±6.5	
50%-75%	30.7±9.9	
Virtually all	33.1±5.0	
^1^Mean±SD of knowledge score ^2^Kruskal-Wallis rank sum test		

**Figure 1:**
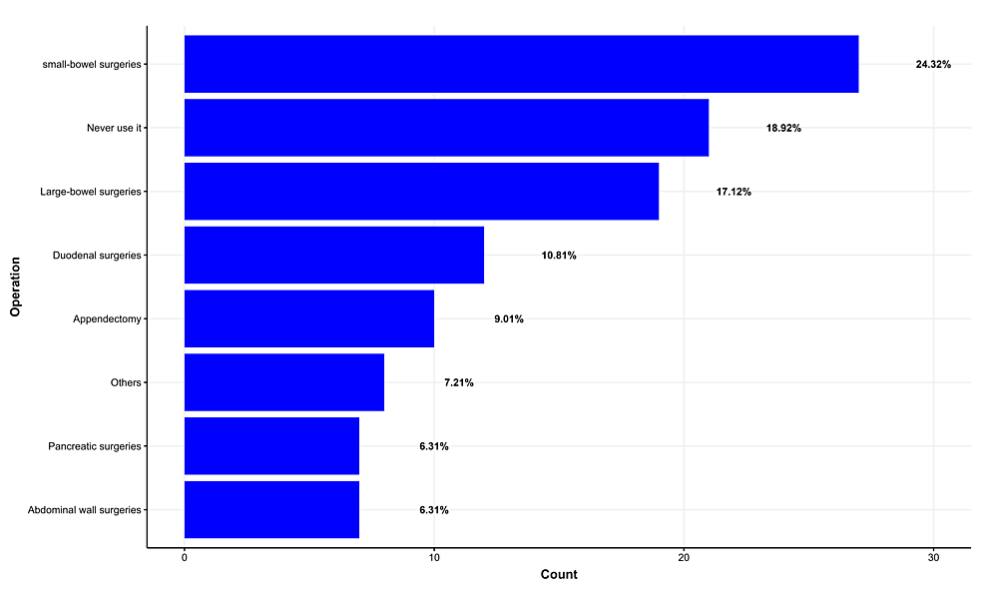
Surgical procedures utilizing anti-adhesive agents

Most surgeons utilize anti-adhesive agents in small bowel surgeries 27 (24.32%), large bowel surgeries 19 (17.12%), duodenal surgeries 12 (10.81%), and appendectomies 10 (9.01%) (Figure 1).

## Discussion

In this study, we aimed to assess the knowledge and clinical practice of anti-adhesive barriers among surgeons in Saudi Arabia. Although the best guarantee against the formation of adhesions is the safe surgical approach, several commercially available anti-adhesive products can be used, including mechanical (barriers) and pharmacological agents. In a case-control study that tested the efficacy of different anti-adhesive products, including chitin layers, methylprednisolone, and hyaluronic-containing products, they that all these products helped decrease adhesion frequency [18,19]. Although most are safe to use, data recommend avoiding using products containing hyaluronic acid directly with anastomosis [20,21].

In our study, the majority disagreed with the notion that “adhesions are not of clinical interest.” The distribution of the item by profession was 48% of the general surgeons, 27% of the trainees, and 13% of the specialized surgeons. These percentages are comparable to the findings of another study conducted in Saudi Arabia by Alruwaili and his colleagues. They found that 19% of the general surgeons didn’t agree that “adhesions are not of clinical interest”; similarly, 18% of the trainees and 10% of the specialized surgeons disagreed [22]. This indicates an increasing awareness of post-operative adhesions and anti-adhesives among surgeons.

The proportion of those who believe in the importance of adhesion prevention was higher among general surgeons 35% than specialized surgeons 24% and trainees 13%. A similar pattern of these responses was reported by Alruwaili et al., where 12% of general surgeons, 6% of specialized surgeons, and 5% of the trainees believed that adhesion prevention is important [22]. In a national survey in Dutch, 67.7% of their participants indicated that adhesions had a negative clinical effect and required attention from surgeons [17]. This difference and higher perception of anti-adhesive importance among general surgeons compared to specialized surgeons might be attributed to the fact that general surgeons deal more with abdominal surgeries where adhesion and leakage are major dilemmas.

Regarding the use of anti-adhesives, 25% of our participants reported never using anti-adhesives in laparotomies or laparoscopic surgeries 38%.

Eighty-five percent of our study participants believe that the use of anti-adhesives will increase. Nevertheless, 38% of gynecologists used anti-adhesions routinely. Yet, another study among gynecologists reported that despite 78.8% of gynecologists being aware of the available anti-adhesive products [23,24], the majority rely on the surgical technique alone as an anti-adhesive measure, such as gentle tissue handling, good hemostasis, and keeping the surgical field moist. Irrigating the surgical field using solutions such as crystalloids or high-molecular-weight dextran has been believed to aid in preventing or reducing post-surgical adhesion formation [25].

A quarter of our participants 25% reported only 5-10% times informing patients about adhesions or adhesion-related morbidity as a possible complication after laparotomy. In the national study of Dutch surgeons, only 9.8% reported informing patients routinely about adhesions in the pre-operative consent, while 40.9% reported never informing patients about adhesions [17]. However, a study among gynecologists demonstrated that 83% informed their patients about the risk of adhesions before surgeries [23]. In a different study among gynecologists in the UK, 61% routinely informed their patients about adhesions [24]. In a cross-sectional study, patients were asked if they were told about adhesions as a possible post-operative complication in the informed consent. This complication was documented in only 8.5% of the pre-operative forms [26].

Although 35% of our participants agreed that adhesion prevention should be applied with specific indications, about three-quarters experienced a lack of clarity about when to use an anti-adhesive agent. Compared with the Alruwaili study, 29.9% of surgeons in their study experienced a lack of certainty about specific anti-adhesive indications. Yet knowledge about anti-adhesive indications increases with experience; specialized surgeons are more knowledgeable than general surgeons [22]. A study found a positive correlation between the uncertainty about indications of anti-adhesives products with never using these products before and lower knowledge about anti-adhesives [17].

Costs of anti-adhesive agents are one of the influential factors in the decision to use these agents by surgeons. In this survey, 41% of participants strongly agreed that “costs do not outweigh the possible benefits of anti-adhesive agents.” Compared to 21.8% of surgeons in the Alruwaili survey who strongly agreed that costs don’t outweigh the benefits, 46% indicated that cost should not be a barrier to using anti-adhesives [22].

In the current study, 42% of surgeons agreed that adhesiolysis is not effective in managing complaints of pain. Nevertheless, the gynecologist agreed to perform adhesiolysis for symptomatic patients only [23]. The national survey about adhesion awareness was re-conducted at a six-year interval, and the authors found no increase in surgeons’ knowledge about adhesions. Yet, the inclusion of adhesions as a possible post-operative complication in the consent was increased [27].

Our study is limited to a sample of Saudi surgeons and trainees, yet its relatively large sample size increases its validity and generalizability.

## Conclusions

Most participants admit that post-operative adhesions are of great clinical interest and could be prevented. Nevertheless, over 25% of respondents stated they had never used anti-adhesive products during laparoscopic procedures or laparotomies. Most surgeons didn’t include adhesions as a post-operative complication in the informed consent. Although about 35% of Saudi surgeons believe in the importance of anti-adhesives, three-quarters experienced a lack of clarity about the specific indications to use an anti-adhesive agent. It is recommended that more educational sessions be conducted during the surgical training period, such as developing guidelines and protocols to use anti-adhesive barriers, providing educational materials for healthcare professionals, and presenting them in courses and conferences, to increase surgeons’ awareness about adhesions as one of the major postoperative complications and to increase the use of different available barriers and pharmacological anti-adhesive products whenever appropriate.

## Appendices

**Table 6:** Awareness of anti-adhesive barriers questionnaire

Are you a surgeon?	Yes
	No
You are:	Trainee
	General surgeon
	Oncologic surgeon
	Vascular surgeon
	Gastrointestinal surgeon
	Pediatric surgeon
	Trauma surgeon
	OB/GYN
	Other
Place of work:	o Write your answer.
How many years of work experience (as a trainee or as a surgeon) do you have:	4-5 Years
	One year or less
	2-3 Years
	More than 5 years
Adhesions are not of clinical interest:	Totally disagree.
	Disagree
	Neutral
	Agree
	Totally agree
Adhesions have more beneficial than detrimental effects:	Totally disagree.
	Disagree
	Neutral
	Agree
	Totally agree
Adhesiolysis for complaints of pain is not effective:	Totally disagree.
	Disagree
	Neutral
	Agree
	Totally agree
How many patients do you inform about adhesions or adhesion related morbidity as a possible complication after laparotomy surgery:	None
	<5%
	5-10%
	10%-25%
	25%-50%
	50%-75%
	Virtually all
How many patients do you inform about adhesions or adhesion related morbidity as a possible complication after laparoscopic surgery:	None
	<5%
	5-10%
	10%-25%
	25%-50%
	50%-75%
	Virtually all
You do not believe in adhesion prevention:	Totally disagree.
	Disagree
	Neutral
	Agree
	Totally agree
You would like to apply adhesion prevention in all abdominal operations:	Totally disagree.
	Disagree
	Neutral
	Agree
	Totally agree
You would like to apply adhesion prevention only in certain indications:	Totally disagree.
	Disagree
	Neutral
	Agree
	Totally agree
Laparoscopic surgery causes fewer adhesions than open surgery:	Totally disagree.
	Disagree
	Neutral
	Agree
	Totally agree
Meticulous surgical technique (tissue handling, avoiding gauzes) reduces adhesions:	Totally disagree.
	Disagree
	Neutral
	Agree
	Totally agree
Extraperitoneal mesh position causes fewer adhesions than the intraperitoneal mesh position:	Totally disagree.
	Disagree
	Neutral
	Agree
	Totally agree
A coated mesh causes fewer adhesions than an uncoated mesh:	Totally disagree.
	Disagree
	Neutral
	Agree
	Totally agree
Electrocautery causes fewer adhesions:	Totally disagree.
	Disagree
	Neutral
	Agree
	Totally agree
Less intraperitoneal suture material reduces adhesions:	Totally disagree.
	Disagree
	Neutral
	Agree
	Totally agree
You don’t believe in antiadhesive agents:	Totally disagree.
	Disagree
	Neutral
	Agree
	Totally agree
You experience a lack of clarity about when to use an antiadhesive agent:	Totally disagree.
	Disagree
	Neutral
	Agree
	Totally agree
You prefer using a locally acting antiadhesive agent over an agent that acts throughout the whole abdomen:	Totally disagree.
	Disagree
	Neutral
	Agree
	Totally agree
You think the costs do not outweigh the possible benefits of antiadhesive agents:	Totally disagree.
	Disagree
	Neutral
	Agree
	Totally agree
Which factors influence your belief in adhesion Prevention:	o Write your answer.
Have you ever used one of these Anti-adhesive agents:	Interceed
	Seprafilm
	Adept
	Spraygel
	Hyalobarrier
	Prevadh
	Ringer’s lactate
	Other
	Never used Anti-adhesive agent
With which operation have you ever used anti-adhesive agents: o Pancreatic surgeries	Duodenal surgeries
	Small-bowel surgeries
	Large-bowel surgeries
	Appendectomy
	Abdominal wall surgeries
	Other: write your response
How many times are anti-adhesive agents in laparotomies used by you or your colleagues in your hospital:	Never used
	One time
	2 times
	3 times
	4 times
	More than 5 times
How many times are anti-adhesive agents in laparoscopic used by you or your colleagues in your hospital:	Never used
	One time
	2 times
	3 times
	4 times
	More than 5 times
Do you believe the use of anti-adhesive Barriers will increase in the future:	Yes
	No
